# Presence and Absence of Muscle Contraction Elicited by Peripheral Nerve Electrical Stimulation Differentially Modulate Primary Motor Cortex Excitability

**DOI:** 10.3389/fnhum.2017.00146

**Published:** 2017-03-24

**Authors:** Ryoki Sasaki, Shinichi Kotan, Masaki Nakagawa, Shota Miyaguchi, Sho Kojima, Kei Saito, Yasuto Inukai, Hideaki Onishi

**Affiliations:** Department of Physical Therapy, Institute for Human Movement and Medical Sciences, Niigata University of Health and WelfareNiigata, Japan

**Keywords:** muscle contraction, somatosensory input, peripheral nerve electrical stimulation, transcranial magnetic stimulation, motor evoked potential, primary motor cortex

## Abstract

Modulation of cortical excitability by sensory inputs is a critical component of sensorimotor integration. Sensory afferents, including muscle and joint afferents, to somatosensory cortex (S1) modulate primary motor cortex (M1) excitability, but the effects of muscle and joint afferents specifically activated by muscle contraction are unknown. We compared motor evoked potentials (MEPs) following median nerve stimulation (MNS) above and below the contraction threshold based on the persistence of M-waves. Peripheral nerve electrical stimulation (PES) conditions, including right MNS at the wrist at 110% motor threshold (MT; 110% MNS condition), right MNS at the index finger (sensory digit nerve stimulation [DNS]) with stimulus intensity approximately 110% MNS (DNS condition), and right MNS at the wrist at 90% MT (90% MNS condition) were applied. PES was administered in a 4 s ON and 6 s OFF cycle for 20 min at 30 Hz. In Experiment 1 (*n* = 15), MEPs were recorded from the right abductor pollicis brevis (APB) before (baseline) and after PES. In Experiment 2 (*n* = 15), M- and F-waves were recorded from the right APB. Stimulation at 110% MNS at the wrist evoking muscle contraction increased MEP amplitudes after PES compared with those at baseline, whereas DNS at the index finger and 90% MNS at the wrist not evoking muscle contraction decreased MEP amplitudes after PES. M- and F-waves, which reflect spinal cord or muscular and neuromuscular junctions, did not change following PES. These results suggest that muscle contraction and concomitant muscle/joint afferent inputs specifically enhance M1 excitability.

## Introduction

Converging evidence suggests that afferent somatosensory inputs such as peripheral nerve electrical stimulation (PES), muscle tendon vibration and active and passive movements can induce changes in primary motor cortex (M1) excitability (Naito et al., 1999, 2002; Ridding et al., 2000; Kaelin-Lang et al., 2002; Macé et al., 2008; Miyaguchi et al., 2013; Onishi et al., 2013; Kotan et al., 2015). Somatosensory inputs play a major role in motor control at the cortical level; this is a critical aspect of sensorimotor integration. For example, human and animal studies have shown reduced sensory function results in decreased manual motor function (Twitchell, 1954; Rothwell et al., 1982; Sanes et al., 1984). In addition, somatosensory input is required for motor learning (Pavlides et al., 1993).

Previous studies have reported changes in motor evoked potentials (MEPs) elicited by transcranial magnetic stimulation (TMS) over the M1 after a prolonged period of PES (Ridding et al., 2000; Fraser et al., 2002; Tinazzi et al., 2005; Chipchase et al., 2011b). These MEP alterations have been proposed depending on the intensity (Chipchase et al., 2011a; Schabrun et al., 2012), frequency (Mang et al., 2010; Golaszewski et al., 2012), and duration (Andrews et al., 2013) of PES. Studies have also shown alterations in TMS-evoked MEPs following PES without concomitant changes in brainstem electrical stimulation-evoked MEPs (Kaelin-Lang et al., 2002) or electrical stimulation-evoked M- and F-waves, or H-reflex (Tinazzi et al., 2005; Mang et al., 2010; Golaszewski et al., 2012), suggesting that the observed modulation occurs at the cortical level. However, the mechanism underlying the alteration in excitability in the M1 after PES remains poorly understood.

Different somatosensory modalities are channeled to specific areas of somatosensory cortex (S1). In animals, S1 areas 3a and 2 predominantly receive proprioceptive inputs from muscle and joint afferents (Rasmusson et al., 1979; Pons et al., 1992; Iwamura et al., 1993), while cutaneous afferent inputs predominantly reach areas 3b and 1 (Jones and Friedman, 1982; Pons et al., 1992; Kaas, 2004). Therefore, different areas of S1 are stimulated depending on whether peripheral nerve stimulation is sufficient to induce muscle contraction. In turn, differential activation of S1 by PES above or below the motor threshold (MT) may have distinct effects on M1 excitability and motor function. Indeed, MEP amplitude increased following PES sufficient to produce muscle contraction (Andrews et al., 2013) but decreased when PES was applied at subthreshold intensities (Mima et al., 2004; Tinazzi et al., 2005). However, these studies do not compare between PES above and below the contraction threshold. One study compared MEPs following PES above and below the contraction threshold and reported that PES eliciting muscle contraction significantly increases MEP amplitude, while sub-MT PES significantly decreases MEP amplitude (Schabrun et al., 2012). However, it is unclear whether the changes in MEP amplitude are due to muscle contraction specifically or simply the electrical stimulation intensity, because Schabrun et al. (2012) used different stimulation intensity between PES conditions (motor stimulation and sensory stimulations) for the same site (abductor pollicis brevis, APB). Therefore, the details of the alterations in excitability in the M1 after PES with and without muscle contraction are unknown. Although PES is a widely used rehabilitation technique for numerous neurological and musculoskeletal disorders, there are no standard protocols for PES conditions (motor and sensory stimulations) such as stimulation intensity, frequency and duty cycle. If the physiological impacts underlying the PES are better understood, PES may be useful in the treatment of patients. Here we aimed to clarify whether the presence and absence of muscle contraction elicited by PES differentially alters M1 excitability using identical PES protocols such as the stimulation frequency, duration and duty cycle. We compared MEPs from TMS following median nerve stimulation (MNS) above and below the contraction threshold for different sites (wrist [mixed nerve] and index finger [sensory nerve]). Three PES conditions were applied: (1) right MNS at the wrist at 110% MT (110% MNS condition); (2) right MNS at the index finger (sensory digit nerve stimulation [DNS]) with stimulus intensity ~110% MNS (DNS condition); and (3) right MNS at the wrist at 90% MT (90% MNS condition). Moreover, 110% MNS and DNS conditions were used to compare the influence of PES with and without muscle contraction on attaining similar PES intensity. A 90% MNS condition was adopted to compare the influence PES at the wrist (mixed nerve) with and without muscle contraction. In fact, we wanted to clarify whether muscle contraction is important for bi-directional MEP changes at the same site (mixed nerve) even if the stimulation intensity is not 110% MNS. We thought that these conditions are necessary to investigate the presence and absence of muscle contraction, stimulation intensity and area of stimulation part for obtaining conclusive evidence on whether muscle is contraction specifically important for bi-directional changes. We hypothesized that the unique S1 activity patterns induced by PES intensities above and below MT would have differential effects on M1 excitability. Specifically, we speculated that PES with muscle contraction would increase M1 excitability, whereas PES without muscle contraction would decrease M1 excitability.

## Materials and Methods

### Subjects

Twenty-six healthy subjects (17 males and 9 females; mean ± standard deviation, 21.7 ± 1.8 years; age range, 20–30 years) participated in this study. Twenty-three subjects were right handed, and three were left handed. Based on health-related and TMS questionnaires (Rossi et al., 2009), none of the subjects had a history of neurological disorders, were taking drugs that affected functioning of the central nervous system, or had contraindications for TMS. All subjects provided written informed consent before participating in the experiment. This study conformed to the guidelines stated in the Declaration of Helsinki and was approved by the ethics committee of the Niigata University of Health and Welfare.

### Electromyography Recordings

Surface electromyography (EMG) was recorded from the right APB muscle via disposable Ag/AgCl electrodes in a belly-tendon montage. The signals from the EMG electrodes were amplified (×100) by an amplifier (A-DL-720-140, 4 Assist, Tokyo, Japan), filtered (high pass, 20 Hz), digitized at 4 kHz using an A/D converter (Power Lab 8/30, AD Instruments, Colorado Springs, CO, USA), and stored on a lab computer for later offline analysis (LabChart7, AD Instruments).

### MEP Recordings from TMS

Magnetic stimuli were delivered through a figure-of-eight coil (diameter, 9.5 cm) connected to a Magstim 200 stimulator (Magstim, Dyfed, UK). The coil was held tangentially to the skull over the left M1 area with the handle pointing posterolaterally at 45° to the sagittal plane. The TMS coil was placed over the left M1 at the position producing the largest MEPs from the right APB muscle (the motor hotspot). Position and orientation of the coil for the motor hotspot were marked according to magnetic resonance imaging (MRI) via Visor2 TMS Neuronavigation (eemagine Medical Imaging Solutions GmbH, Berlin, Germany), and the coil was held in place to maintain position. T1-weighted MRI was obtained using a 1.5-T system before the experiment (Signa HD, GE Healthcare, Milwaukee, WI, USA). The TMS intensity was set at that inducing peak-to-peak amplitude of ~1 mV in APB at baseline. The TMS intensity is expressed as a percentage of the maximum stimulator output.

### Peripheral Nerve Electrical Stimulation

Continuous electrical stimulation was delivered through bar electrodes to the right median nerve at the wrist (mixed nerve) or the right index finger tip (sensory nerve) by means of an electrical generator (SEN-8203, Nihon Kohden, Tokyo, Japan). The electrical stimulation (0.2-ms square wave constant current pulses) was delivered for 20 min at 30 Hz in a 4 s ON and 6 s OFF cycle in all PES conditions. The MT was determined based on the minimum stimulus intensity that can elicit M-waves (i.e., the minimum stimulus intensity that can observe the persistence of M-waves) to the right median nerve at the wrist (mixed nerve) using surface EMG. Three different PES conditions were used: (1) electrical stimulation to the right median nerve at the wrist with intensity set to 110% MT (110% MT/mixed nerve electrical stimulation, 110% MNS); (2) electrical stimulation to the right index finger tip with intensity equal to that used in condition (1) DNS; and (3) electrical stimulation to the right median nerve at the wrist with intensity at 90% MT (90% MT/mixed nerve electrical stimulation, 90% MNS). All subjects felt tactile perception without pain in DNS condition.

### M- and F-wave Recordings

Electrical pulses (0.2-ms square wave constant current pulses) were delivered through bar electrodes to the right median nerve at the wrist using an electrical generator (SEN-8203, Nihon Kohden, Tokyo, Japan). M- and F-waves were recorded from right APB using surface EMG. Stimulus intensity was 120% of the stimulus strength required to produce maximum M-wave.

### Experimental Conditions

**Figure 1:**
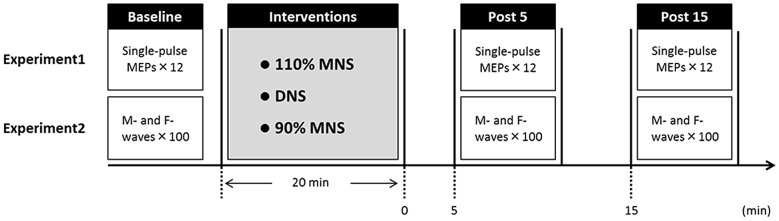
**Experimental protocol.** Fifteen subjects each participated in Experiments 1 and 2 examining the effects of three PES conditions (110% MNS, DNS and 90% MNS) on transcranial magnetic stimulation (TMS)-induced motor evoked potentials (MEPs) and PES-evoked M-waves and F-waves, respectively. The minimum period between sessions for a single subject was 3 days. In Experiment 1, MEPs were recorded 12× before PES (baseline) and both 5 min (post 5) and 15 min (post 15) after PES. In Experiment 2, M- and F-waves were recorded 100× before PES (baseline) and both 5 min (post 5) and 15 min (post 15) after PES. MNS, median nerve stimulation; DNS, digit nerve stimulation; PES, peripheral nerve electrical stimulation.

Fifteen healthy subjects (10 males and 5 females; mean ± standard deviation, 22.1 ± 2.2 years; age range, 20–29 years) participated in Experiment 1. The experimental protocols are shown in Figure 1. Subjects were seated in a comfortable reclining chair with a mounted headrest during experiments. Each subject was subjected to the three PES conditions (110% MNS, DNS and 90% MNS) on separate days at least 3 days apart. In Experiment 1, 12 MEPs each were recorded with an interstimulus interval of 5 s before (baseline) and at 5 and 15 min after PES (post 5 and post 15 time points, respectively). The same TMS intensity was used before and after PES interventions.

Fifteen healthy subjects (10 males and 5 females; mean ± standard deviation, 22.3 ± 2.4 years; age range, 20–30 years) participated in Experiment 2. We conducted Experiments 1 and 2 on different days, and the subjects who participated in our study were different for different experiments. Hundred M- and F-waves were recorded with an interstimulus interval of 1 s before (baseline) and 5 and 15 min after PES (post 5 and post 15). The same electrical stimulus intensity was used before and after PES.

In these experiments, DNS condition was performed after 110% MNS condition to decide on DNS intensity based on the 110% MNS intensity. Thus, these experiments were conducted in the following order to avoid the effects pertaining to the sequence: 110% MNS, DNS and 90% MNS; 110% MNS, 90% MNS and DNS; or 90% MNS, 110% MNS and DNS.

### Data Analysis and Statistics

For Experiment 1, the peak-to-peak amplitudes of 10 of the 12 recorded MEPs (excluding the maximum and minimum) were averaged for each time point (baseline, post 5 and post 15; Miyaguchi et al., 2013; Kojima et al., 2015). For Experiment 2, the peak-to-peak amplitudes of M-waves and F-waves were averaged at each time point (baseline, post 5 and post 15). F-waves are also expressed as a percentage of maximum M-wave amplitude (i.e., F/M ratio). F-wave persistence was calculated based on the F-wave amplitude that elicited an EMG response of more than 50 μV. F-wave persistence is the number of F-waves present given a specific criterion. Statistical analysis was performed using PASW statistics software version 21 (SPSS; IBM, Armonk, NY, USA). One-way repeated-measures analysis of variance (ANOVA) was used to compare the main effects of SESSION on TMS intensity. Two-way repeated-measures ANOVA was used to compare the main effects of INTERVENTION (PES 110% MNS, DNS, 90% MNS) and TIME (baseline, post 5 and post 15) and their interaction (INTERVENTION × TIME) on MEP amplitudes, M-wave amplitudes, F-wave amplitudes, F/M ratios and F-wave persistence. The Mauchly’s test of sphericity was used to evaluate the sphericity assumption. If the Mauchly’s test of sphericity assumption was violated, the Greenhouse–Geisser correction was used to adjust the significant values. When a significant main effect or interaction was found, Bonferroni test was used for *post hoc* comparisons. Also, unpaired *t*-test was used to compare PES intensity between PES conditions in Experiments 1 and 2. Statistical significance for all tests was set at *P* < 0.05.

## Results

### Electrical Stimulus Intensity

In Experiment 1, the mean PES current amplitudes were 10.3 ± 0.6 mA for 110% MNS, 10.3 ± 0.6 mA for DNS, and 8.1 ± 0.6 mA for 90% MNS. In the Experiment 2, the mean currents for these PES conditions were 8.7 ± 0.6 mA, 8.7 ± 0.6 mA and 7.9 ± 0.7 mA, respectively. There were no significant differences in the PES intensity between experiments in each of the PES conditions (110% MNS conditions, *P* = 0.080; DNS conditions, *P* = 0.080; 90% MNS conditions, *P* = 0.890; unpaired *t*-test).

### Experiment 1: Effects of PES on MEP Amplitudes

The TMS intensity was 56.8% ± 1.9% of maximum stimulator output in the 110% MNS PES condition, 57.1% ± 1.9% in the DNS condition, and 57.3% ± 1.9% in the 90% MNS condition. One-way repeated-measures ANOVA showed no significant effects of SESSION (*F*_(2,28)_ = 0.164, *P* = 0.849) on the TMS intensity.

**Figure 2:**
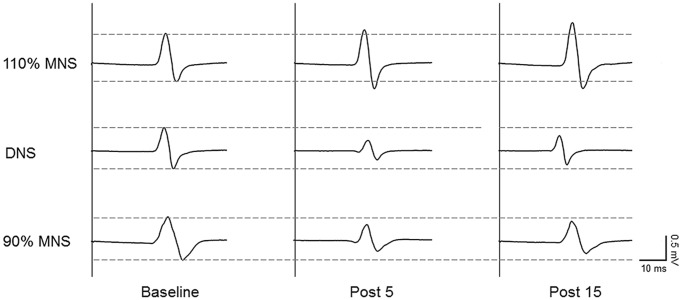
**Representative data of changes in MEPs before and after PES conditions.** Representative 10-trial averaged MEPs measured in the right abductor pollicis brevis (APB) muscle before (baseline, left) and after (right) the three PES protocols: 110% of MT/mixed MNS (110% MNS), DNS at 110% MNS (DNS) and 90% MT/mixed MNS (90% MNS). The MEP amplitude increased for 15 min after PES to the mixed nerve eliciting muscle contraction. In contrast, the MEP amplitude decreased for 15 min after PES to the sensory and mixed nerves in the absence of muscle contraction. MEP, motor evoked potential; PES, peripheral nerve electrical stimulation; MNS, median nerve stimulation; MT, motor threshold.

Sample MEP waveforms recorded from a representative subject in each PES condition (Figure 2) demonstrate our basic findings; application of 110% MNS increased the MEP amplitude in response to constant TMS output (top row), indicating enhanced M1 excitability, while both DNS and 90% MNS (below MT) reduced MEP amplitude (bottom rows). Two-way repeated-measures ANOVA revealed a significant main effect of INTERVENTION (*F*_(2,28)_ = 14.147, *P* < 0.001, *η*^2^ = 0.503) and an INTERVENTION × TIME interaction (*F*_(4,56)_ = 9.478, *P* < 0.001, *η*^2^ = 0.404) but no significant main effect of TIME (*F*_(2,28)_ = 3.137, *P* = 0.059, *η*^2^ = 0.183). Baseline MEP amplitudes did not differ between PES conditions (110% MNS vs. DNS, *P* = 1.000; DNS vs. 90% MNS, *P* = 1.000; 110% MNS vs. 90% MNS, *P* = 1.000). At each post-PES time point (post 5 and post 15), *post hoc* tests revealed a significant difference in MEP amplitude between 110% MNS and both DNS and 90% MNS conditions (post 5: 110% MNS vs. DNS, *P* = 0.001; 110% MNS vs. 90% MNS, *P* < 0.001; post 15: 110% MNS vs. DNS, *P* = 0.001; 110% MNS vs. 90% MNS, *P* < 0.001) but no difference between DNS and 90% MNS conditions (post 5: *P* = 1.000; post 15: *P* = 1.000; Table 1).

**Table 1:** **Results of the motor evoked potentials (MEPs) and M- and F-waves for three peripheral nerve electrical stimulation (PES) conditions**.

	Baseline	Post 5	Post 15
**MEP amplitudes (mV)**
110% MNS	1.00 ± 0.04	1.26 ± 0.09*	1.26 ± 0.12*
DNS	0.98 ± 0.03	0.68 ± 0.11*^†^	0.77 ± 0.07*^†^
90% MNS	0.98 ± 0.03	0.66 ± 0.07*^†^	0.68 ± 0.08*^†^
**M-wave amplitudes (mV)**
110% MNS	18.8 ± 0.9	18.8 ± 0.9	18.9 ± 1.0
DNS	18.4 ± 1.0	17.7 ± 1.0	18.0 ± 1.0
90% MNS	19.4 ± 1.3	19.6 ± 1.3	19.7 ± 1.3
**F-wave amplitudes (mV)**
110% MNS	0.30 ± 0.03	0.32 ± 0.04	0.30 ± 0.04
DNS	0.31 ± 0.04	0.28 ± 0.03	0.28 ± 0.03
90% MNS	0.37 ± 0.04	0.36 ± 0.04	0.38 ± 0.04
**F/M ratio (%)**
110% MNS	1.58 ± 0.14	1.71 ± 0.20	1.63 ± 0.18
DNS	1.74 ± 0.19	1.63 ± 0.19	1.58 ± 0.17
90% MNS	1.92 ± 0.21	1.85 ± 0.17	1.94 ± 0.17
**F-wave persistence (%)**
110% MNS	55.1 ± 6.1	51.1 ± 6.1	49.1 ± 6.3
DNS	56.7 ± 5.9	52.3 ± 6.0	52.2 ± 6.3
90% MNS	60.5 ± 5.0	60.1 ± 5.3	63.5 ± 5.4

All data are expressed as mean ± SE. 110% MNS, 110% of motor threshold/mixed median nerve stimulation; DNS, digit nerve stimulation; 90% MNS, 90% of motor threshold/mixed median nerve stimulation, *P < 0.05 baseline vs. post 5 and post 15. ^†^*P* < 0.01 110% MNS vs. DNS and 90% MNS.

**Figure 3:**
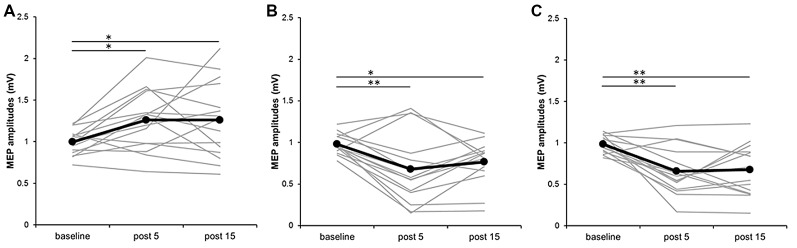
**Effects of presence and absence of muscle contraction induced by PES on MEP amplitudes in individual subjects. (A)** 110% MNS, **(B)** DNS and **(C)** 90% MNS. Black lines show the mean MEP amplitudes recorded before (baseline) PES and 5 min after (post 5) and 15 min after (post 15) PES (*n* = 15). Gray lines show the individual MEP amplitude at each time point. 110% MNS (mixed nerve stimulation) eliciting muscle contraction increased MEP amplitudes at post 5 and post 15 vs. baseline (baseline vs. post 5, *P* = 0.048; baseline vs. post 15, *P* = 0.049). In contrast, DNS (sensory nerve stimulation) and 90% MNS (mixed nerve stimulation) on the absence of muscle contraction decreased MEP amplitudes at post 5 and post 15 vs. baseline (DNS: baseline vs. post 5, *P* = 0.002; baseline vs. post 15, *P* = 0.032; 90% MNS: baseline vs. post 5, *P* < 0.001; baseline vs. post 15, *P* = 0.001). *P < 0.05 and **P < 0.01. MNS, median nerve stimulation; DNS, digit nerve stimulation; MEP, motor evoked potential; PES, peripheral nerve electrical stimulation.

Figure 3 plots the individual MEP data from all subjects for the three different PES conditions. In the 110% MNS condition, *post hoc* tests showed a significant increase in MEP amplitude at post 5 and post 15 compared with baseline (baseline vs. post 5, *P* = 0.048; baseline vs. post 15, *P* = 0.049). Alternatively, in both the DNS and 90% MNS conditions, *post hoc* tests showed a significant decrease in MEP amplitude at post 5 and post 15 time points compared with baseline (DNS: baseline vs. post 5, *P* = 0.002; baseline vs. post 15, *P* = 0.032; 90% MNS: baseline vs. post 5, *P* < 0.001; baseline vs. post 15, *P* = 0.001; Table 1).

### Experiment 2: Effects of PES on M- and F-waves

**Figure 4:**
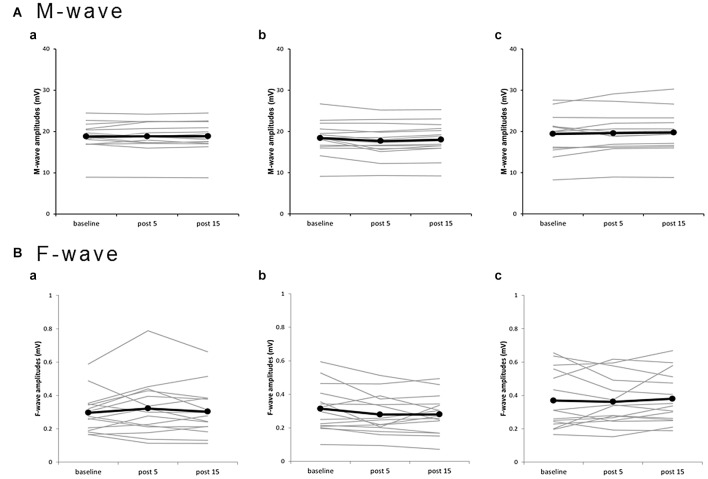
**Effects of presence and absence of muscle contraction induced by PES on M- and F-waves for individual subjects. (a)** 110% MNS, **(b)** DNS, **(c)** 90% MNS. **(A)** M-wave amplitudes. **(B)** F-wave amplitudes. Black lines show the mean amplitudes of M- and F-waves recorded before PES (baseline), 5 min after PES (post 5), and 15 min after PES (post 15; *n* = 15). Gray lines show the individual amplitudes of M- and F-waves recorded at each time point. No significant changes in M- and F-waves, which reflect spinal cord or muscular and neuromuscular junction, were observed after PES. MNS, median nerve stimulation; PES, peripheral nerve electrical stimulation.

Plots of individual M-wave amplitudes (Figure 4A) and F-wave amplitudes (Figure 4B) from all subjects for the three PES conditions suggest no significant changes. Two-way repeated-measures ANOVA showed no significant main effects of INTERVENTION (*F*_(2,28)_ = 2.182, *P* = 0.132, *η*^2^ = 0.135) and TIME (*F*_(1.097,15.356)_ = 0.823, *P* = 0.389, *η*^2^ = 0.056) and no INTERVENTION × TIME interaction (*F*_(2.102,29.420)_ = 2.260, *P* = 0.120, *η*^2^ = 0.139) on M-waves (Table 1). Similarly, two-way repeated-measures ANOVA showed no significant main effects of INTERVENTION (*F*_(1.417,19.839)_ = 3.000, *P* = 0.087, *η*^2^ = 0.176) and TIME (*F*_(2,28)_ = 0.242, *P* = 0.787, *η*^2^ = 0.017) and no INTERVENTION × TIME interaction (*F*_(4,56)_ = 1.554, *P* = 0.199, *η*^2^ = 0.100) on F-waves. For the F/M ratio, two-way repeated-measures ANOVA showed no significant main effects of INTERVENTION (*F*_(1.357,18.998)_ = 1.943, *P* = 0.178, *η*^2^ = 0.122) and TIME (*F*_(2,28)_ = 0.178, *P* = 0.838, *η*^2^ = 0.013) and no INTERVENTION × TIME interaction (*F*_(4,56)_ = 1.505, *P* = 0.213, *η*^2^ = 0.097). For F-wave persistence, two-way repeated-measures ANOVA showed significant main effects of INTERVENTION (*F*_(2,28)_ = 5.060, *P* = 0.013, *η*^2^ = 0.265), but no significant main effects of TIME (*F*_(2,28)_ = 1.164, *P* = 0.327, *η*^2^ = 0.077) and no INTERVENTION × TIME interaction (*F*_(2.198,30.777)_ = 1.323, *P* = 0.283, *η*^2^ = 0.086; Table 1). *Post hoc* tests conducted for studying the main effect of INTERVENTION revealed a significant difference in F-wave persistence between DNS and 90% MNS conditions (*P* = 0.048) but no difference between 110% MNS and both DNS and 90% MNS conditions (110% MNS vs. DNS, *P* = 1.000; 110% MNS vs. 90% MNS, *P* = 0.077).

## Discussion

This study demonstrates that while mixed nerve electrical stimulation at an intensity above MT (muscle contraction elicited by the stimulation) significantly increases MEP amplitude, indicative of enhanced M1 excitability, the mixed nerve electrical stimulation at an intensity below MT (muscle contraction not elicited by the stimulation) or sensory nerve electrical stimulation decreases MEP amplitude, indicative of reduced M1 excitability. Neither M- nor F-wave amplitude differed between these stimulus conditions, suggesting that the changes occur at the level of the cortex. These results suggested that proprioceptive inputs induced by both mixed nerve electrical stimulation and muscle contraction caused by the electrical stimulation might be involved in the increase in M1 excitability after PES.

Previous studies also reported increased MEP amplitude following PES intensities sufficient to produce muscle contraction (Ridding et al., 2000; Fraser et al., 2002; Andrews et al., 2013) and decreased MEP amplitude when PES was applied at intensities below MT (Mima et al., 2004; Tinazzi et al., 2005; Murakami et al., 2007). However, it was unclear whether the MEP change depended specifically on the presence or absence of muscle contraction or only on the electrical stimulation intensity. Our results indicate that MEP amplitudes are increased by mixed nerve stimulation with muscle contraction and decreased by sensory stimulation without muscle contraction even when the stimulus is above MT (DNS condition), suggesting that afferent inputs specifically related to muscle contraction enhanced M1 excitability.

Cutaneous, muscle and joint afferent inputs are thought to be activated by electrical stimulation of mixed peripheral nerve sufficient to evoke muscle contraction. In contrast, only cutaneous afferent inputs are activated by sensory stimulation to the index finger tip (DNS condition). Animal studies have shown that cutaneous afferent inputs project mainly to areas 3b and 1 (Jones and Friedman, 1982; Pons et al., 1992; Kaas, 2004), while proprioceptive inputs from muscle and joint afferents project mainly to areas 3a and 2 (Rasmusson et al., 1979; Pons et al., 1992; Iwamura et al., 1993). Additionally, several studies reported that muscle afferent inputs also reach M1 (Lucier et al., 1975; Zarzecki et al., 1978). Multiple cortical imaging techniques, including magnetoencephalography, functional MRI and positron emission tomography, have shown that electrical stimulation without muscle contraction and mechanical tactile stimulation to the index finger predominantly activates S1 (Xiang et al., 1997; Terumitsu et al., 2009), whereas motor-point stimulation with contraction of the extensor indicis muscle or passive finger movement activates both M1 and S1 (Weiller et al., 1996; Xiang et al., 1997; Nelles et al., 1999; Radovanovic et al., 2002; Terumitsu et al., 2009; Onishi et al., 2011, 2013). These results provide evidence that different brain regions are activated by mixed nerve stimulation with muscle contraction and sensory nerve stimulation to the index finger without muscle contraction. Therefore, these differences might be involved in the bi-directional MEP changes in our results. In other words, the proprioceptive inputs induced by both mixed nerve electrical stimulation and muscle contraction elicited by the electrical stimulation may have contributed to the increase in MEP after PES.

Mixed nerve electrical stimulation at an intensity above MT (muscle contraction elicited by PES) increased MEP, whereas that at an intensity below MT (muscle contraction not elicited by PES) decreased MEP. One possible explanation for PES-induced MEP facilitation is the specific timing of direct Ia fiber activation by PES and somatosensory input from contracting muscles. Saito et al. (2015) reported MEP potentiation and cortical inhibitory circuit depression following 10 Hz paired-pulse electrical stimulation with 5-ms inter-pulse intervals to the median nerve (mixed nerve) even when stimulus intensity was 80% of MT (i.e., without muscle contraction). Therefore, in the mixed nerve stimulation with muscle contraction condition, M1 excitability may increase if somatosensory inputs from Ia fibers induced by direct electrical stimulation and somatosensory inputs from muscle contraction arrive with a time difference of ~5 ms. On the other hand, Chipchase et al. (2011a) showed that the MNS with intensity of muscle twitch at a frequency of 10 Hz did not change, although muscle contraction occurred. It is considered that tetanic contraction induces a greater change in corticomotor excitability than does a muscle twitch. Although a systematic review showed that MEP changes following PES depends on stimulation frequency (Chipchase et al., 2011b), the optimal PES frequency is yet to be elucidated. In our study, bi-directional MEP changes were observed in three PES conditions, eliciting the presence or absence of muscle contraction at the same frequency (30 Hz). Thus, bi-directional MEP changes may be important to the presence and absence of muscle contraction elicited by PES as well as stimulation frequency. However, it is unknown whether similar MEP changes can be observed with other higher or lower frequencies; therefore, further study is necessary to confirm whether other frequencies can also show similar neuroplastic changes.

Sensory nerve and mixed nerve stimulation did not evoke muscle contraction and decreased MEP in this study. Ridding et al. (2000) reported that PES not evoking muscle contraction to fingers 4 and 5 did not influence MEP in the recorded muscle; however, they enrolled only four subjects and used PES paradigms that are different from our methods (e.g., different stimulation intensity, frequency and duration). On the other hand, several studies have shown that PES not evoking muscle contraction decreased MEP (Mima et al., 2004; Tinazzi et al., 2005; Murakami et al., 2007; Schabrun et al., 2012). MEP depression after sensory nerve and mixed nerve stimulation not evoking muscle contraction in our results are consistent with those of previous reports (Mima et al., 2004; Tinazzi et al., 2005; Murakami et al., 2007; Schabrun et al., 2012).

Experiment 2 was designed to test whether the effect of PES on MEP amplitude was due to excitability changes in cortical, spinal cord, or muscle and neuromuscular junction. No significant changes in M- and F-waves were observed following any of the three PES protocols, consistent with previous studies showing that M-, F- and H-waves were not modified by PES (Ridding et al., 2000; Tinazzi et al., 2005; Mang et al., 2010; Golaszewski et al., 2012). Thus, the changes in MEP amplitude are unlikely due to altered excitability in muscle, at neuromuscular junctions, or in spinal cord. We conclude that the effects of PES on MEP amplitude are most likely produced by changes in cortical excitability. Although a significant difference in the F-wave persistence was observed between interventions, there was no significant interaction between the main effect (TIME) and INTERVENTION × TIME. Therefore, these results showed F-wave persistence did not change due to PES.

This study has several limitations. We conducted Experiments 1 and 2 on different days, with both experiments consisting of a different set of subjects; thus, we cannot compare M1 excitability changes in Experiments 1 and 2 and determine if the differences were significant; large-scale future studies examining the inter- and intra-individual variability in the MEP response following PES are warranted. In addition, S1 excitability changes may contribute to M1 excitability changes because sensory afferents arrive S1 and project S1 to M1. Thus, we also need to examine S1 excitability following PES using somatosensory-evoked potentials (SEPs) for clarifying whether inter- and intra-individual variability in the MEP response related to SEP response reflected S1 excitability. The specific neuroplastic mechanism for PES with and without muscle contraction effects on M1 excitability is unknown. However, different brain activities may contribute to M1 excitability increase and decrease from our results. Studies employing multiple methodologies (e.g., paired-pulse TMS using short-interval intracortical inhibition and intracortical facilitation, or SEPs to examine S1 excitability) are planned to elucidate the underlying neuroplastic mechanism induced by PES with and without muscle contraction. Although we compared the effects of mixed nerve stimulation with those of DNS at the same PES intensity, the number and diameter of neural fiber activated by PES were different. We would, therefore, like to explore whether similar MEP depression can be observed even if a wider range of sensitive nerve branches, including those of the middle finger, are stimulated near future.

In summary, this study demonstrates that mixed MNS eliciting muscle contraction significantly increases MEP amplitude, indicative of enhanced M1 excitability, while sensory MNS alone and sub-MT mixed MNS significantly decrease MEP, indicating reducing M1 excitability. Indeed, the lack of effect on M- and F-waves confirms that this modulation is at the level of the cortex rather than spinal cord, neuromuscular junction, or muscle. This study shows that sensory inputs specific to muscle contraction can enhance M1 excitability.

## Author Contributions

HO and RS conceived the study and designed the experiments. RS and MN conducted the experiments. SM and SKojima performed interpretation of data. RS and SKotan performed the statistical analysis. KS and YI helped writing the manuscript. HO and RS wrote the manuscript. All authors read and approved the final manuscript.

## Conflict of Interest Statement

The authors declare that the research was conducted in the absence of any commercial or financial relationships that could be construed as a potential conflict of interest.
